# Zoonotic Rat Lungworm *Angiostrongylus cantonensis* in Black Rats, Houston, Texas, 2024

**DOI:** 10.3201/eid3109.251710

**Published:** 2025-09

**Authors:** Daniela A. Sierra, Tiana L. Sanders, Erin E. Edwards, Christine M. Molter, Guilherme G. Verocai

**Affiliations:** Texas A&M University College of Veterinary Medicine and Biomedical Sciences, College Station, Texas, USA (D.A. Sierra, T.L. Sanders, G.G. Verocai); Texas A&M Veterinary Medicinal Diagnostic Laboratory, College Station (E.E. Edwards); Houston Zoo, Houston, Texas, USA (C.M. Molter)

**Keywords:** Rat lungworm, *Angiostrongylus cantonensis*, black rats, *Rattus rattus*, Texas, zoonoses, parasites, United States

## Abstract

The *Angiostrongylus cantonensis* rat lungworm is a zoonotic nematode that infects several rat species. This nematode causes eosinophilic meningitis and meningoencephalitis in humans and other accidental hosts. We found a 20% prevalence of *A. cantonensis* lungworms in black rats from a zoo facility in Houston, Texas, USA.

The *Angiostrongylus cantonensis* rat lungworm (Strongylida: Metastrongyloidea) is a widely distributed zoonotic parasitic nematode (*1*). This nematode has an indirect life cycle, requiring a rodent definitive host and a gastropod intermediate host (*2*). The cycle begins when a rat within the genus *Rattus* ingests a gastropod intermediate host infected with third-stage larvae (L3). L3 penetrate the intestinal wall, migrate to the brain, molt twice, and then migrate to the right ventricle and pulmonary arteries, where they develop into adults. Within pulmonary arteries, adults reproduce sexually and female worms lay eggs, which hatch into first-stage larvae (L1) that are subsequently coughed up and swallowed. L1 travel through the gastrointestinal tract and are passed in the feces. L1 then reenters the gastropod either orally or by actively penetrating its foot. L1 molt twice within the gastropod host to develop into infective L3. The L3 may be ingested by paratenic hosts, remaining dormant but infective. 

Accidental hosts, including humans, can become infected through ingestion, deliberately or accidentally, of infected gastropods, paratenic hosts, or L3 (*1*). In those hosts, *A. cantonensis* infection causes eosinophilic meningitis or meningoencephalitis (i.e., neural angiostrongyliasis). Disease in humans is characterized by nonspecific neurologic signs such as neck pain and stiffness and sensitivity to touch and light and may be severe or fatal, particularly without timely or effective intervention (*3*). The first human case of eosinophilic meningitis caused by *A. cantonensis* in the United States occurred in Hawaii (*4*). In addition to travel-related cases (*1*), autochthonous cases of *A. cantonensis* in humans and captive and free-ranging wildlife in the United States have occurred in Alabama, Louisiana, Oklahoma, Mississippi, Florida, Texas, Tennessee, and Georgia (*1*,*4*–*8*).

Finding only a few reported cases of rat lungworm infection in humans and nonhuman primates in Texas (*6*,*9*), and noting a lack of research investigating rodent definitive hosts in the state, we investigated the prevalence of *A. cantonensis* lungworms in rodents captured from a zoo located in the metropolitan area of Houston, Texas, USA. We confirmed autochthonous *A. cantonensis* infections in black rats (*Rattus rattus*) through necropsy, gross and histopathological evaluation, microscopy of nematode specimens, and molecular testing as described previously (*5*) (Appendix Table). 

During March−June 2024, we collected rodents at the Houston Zoo in Harris County, Texas (29.7158° N; 95.3903° W). Of the rats examined, we found 15 (20%) of 75 to be infected with *A. cantonensis* worms. The average number of nematodes per rat was 26.6 (range 2–108. We traced infected rats to groups collected during April−June (Appendix Figure). Of the 15 rats histologically confirmed as infected, 11 showed verminous pneumonia with high larval and egg loads, 11 had cross-sections of adults within pulmonary vessels or the right heart ventricle, and 2 had meningitis due to parasitic larval migration (Figure 1). In 13 of the 15 infected rats, we found eggs or larvae with the characteristic dorsal-spined L1 in the lung sediment. We found no larvae in the 2 remaining rats, coincidentally the 2 with meningitis, compatible with prepatent infections. Histologic examination revealed eosinophilic meningitis in the brain tissue, caused by *A. cantonensis* larvae, which we inferred to be L3, L4, or L5 on the basis of infection progression (Figure 1, panel D). We noted adult specimens and larvae in the pulmonary arteries in association with severe, chronic granulomatous pneumonia.

**Figure 1:**
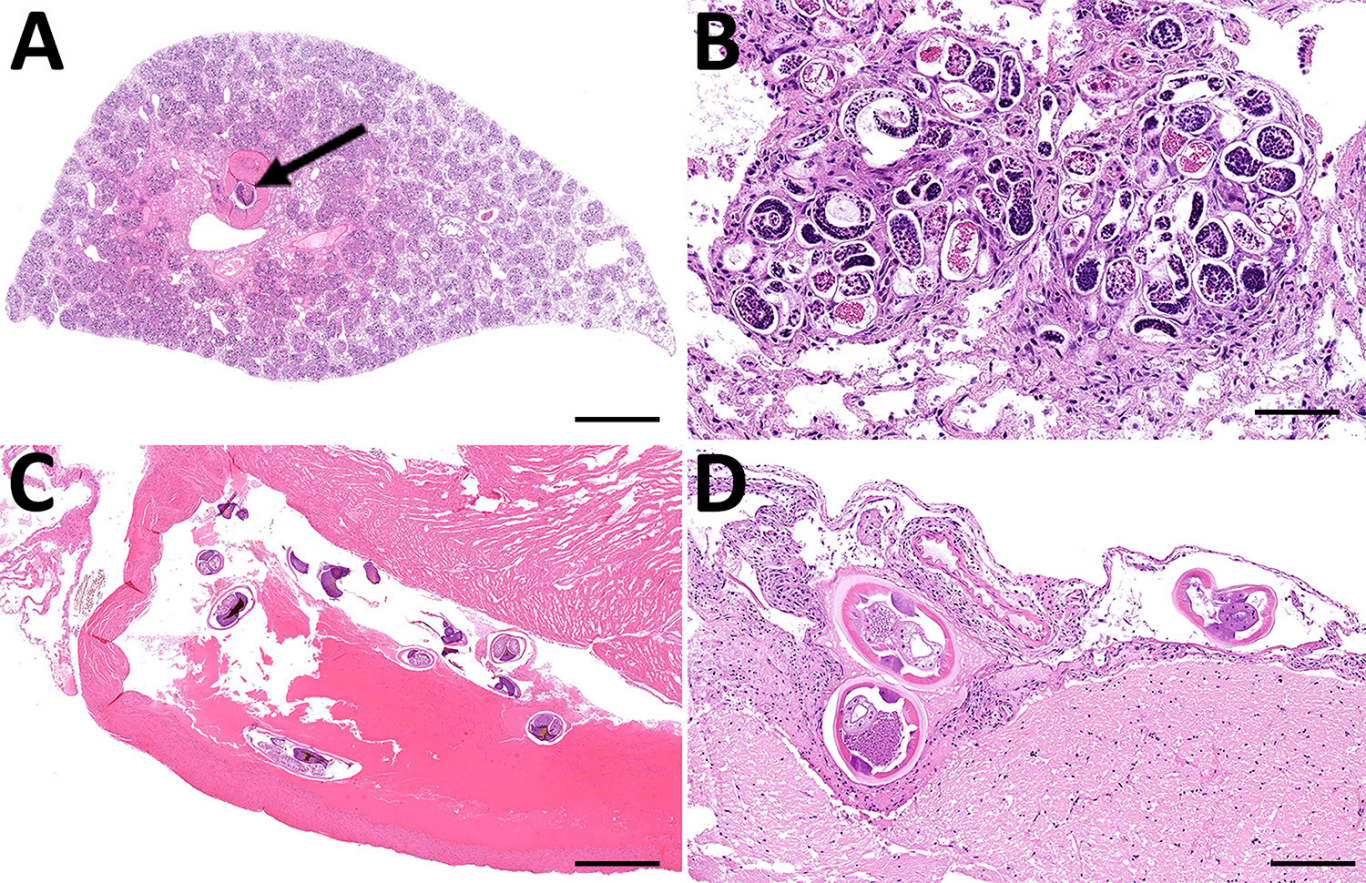
Histopathology of *Angiostrongylus cantonensis* rat lungworm infection in metropolitan black rats, Houston, Texas, 2024. A) Severe pulmonary consolidation due to verminous pneumonia. Adult nematode is visible within a large artery (arrow). Scale bar = 1.5 mm. B) Higher magnification of lung with numerous *A. cantonensis* larvae and eggs surrounded by granulomatous inflammation. Scale bar = 80 µm. C) Multiple adult *A. cantonensis* nematodes in right ventricle of the heart. Scale bar = 1.5 mm. D) Mild lymphoplasmacytic meningitis with cross sections of *A. cantonensis* nematodes. Scale bar = 300 µm.

Our molecular analysis confirmed the identity of each specimen as *A. cantonensis*. The sequences obtained were 100% identical to each other (Figure 2). We submitted 28 sequences that were 190-bp to GenBank (accession nos. PQ556202–29). A 20% prevalence of in the wild black rat population indicates that this parasite is well established at the zoo. We theorize that the parasite is also likely established in the city of Houston and Harris County, the third most populous county in the United States. Studies reported similar prevalence of the nematode in Florida (22.8%) (*7*) and notably higher prevalence in Louisiana (38%) (*8*) and eastern Hawaii Island (93.9%) (*10*). Our results suggest the need for a temporally and geographically broader study to assess parasite distribution and epidemiology in Texas.

**Figure 2:**
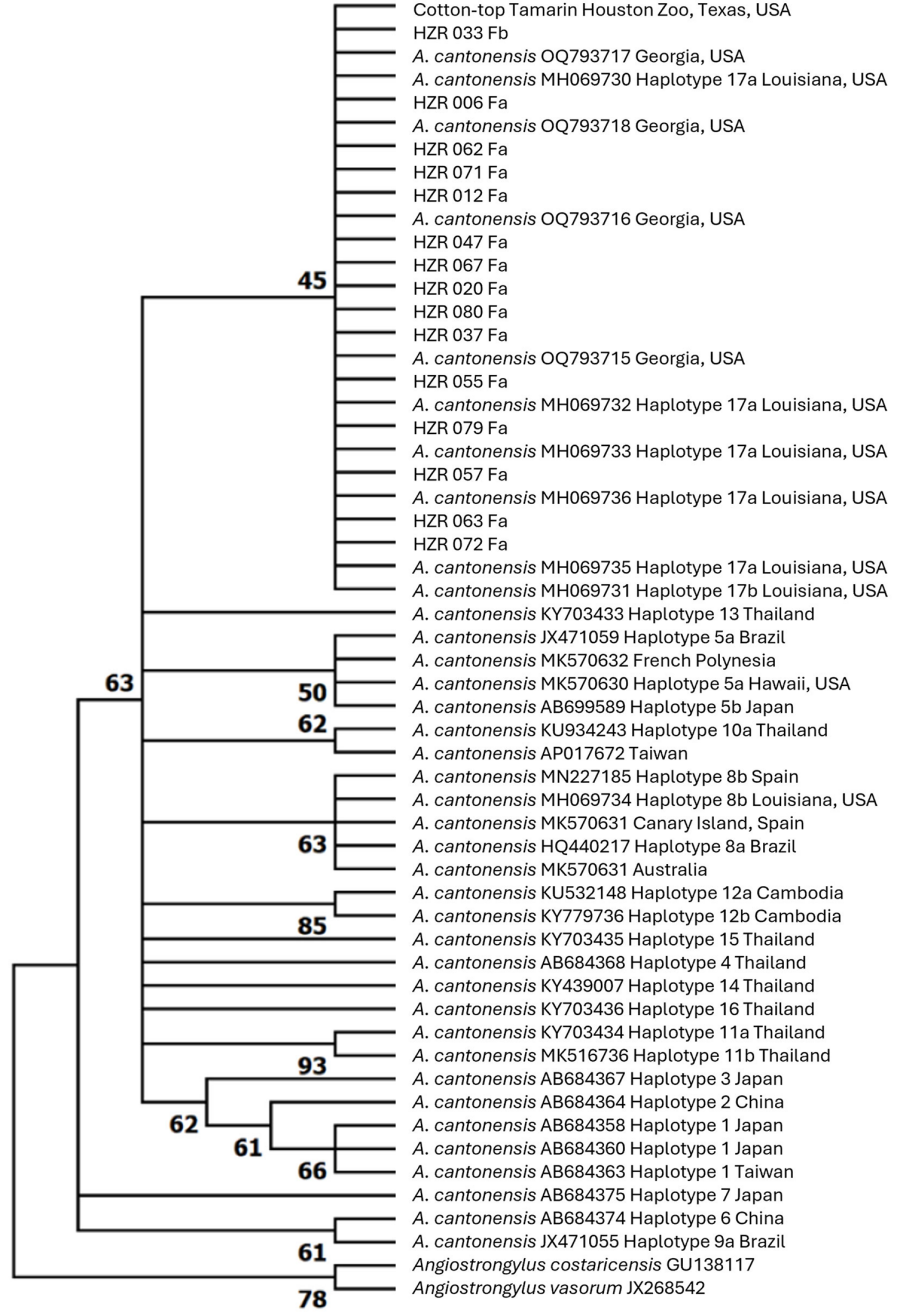
Maximum-likelihood phylogenetic tree (1,000 bootstrap replicates) from study of zoonotic rat lungworm *Angiostrongylus cantonensis* in black rats, Houston, Texas, 2024. Tree depicts the phylogenetic relationships of *A. cantonensis* sequences generated from samples in this study and representative sequences of *A. cantonensis* haplotypes from the United States and globally. Sequences generated in the study are labeled HZR and were deposited into GenBank (accession nos. PQ556202–29). Reference sequences are identified by GenBank accession number and location.

The established *A. cantonensis* cycle within this metropolitan area highlights the risk of zoonotic exposure to humans. In addition, *A. cantonensis* lungworms may be an emerging threat to conservation of threatened or endangered captive animals housed in zoos in endemic areas. The loss of a single animal can have a massive impact on the genetic pool. Therefore, establishment of this nematode in the area imparts greater risk for those endangered species (*6*). The sequences we generated were 100% identical to haplotype 17a, previously found in Louisiana and Georgia (*5*,*8*). This finding suggests that, after introduction and establishment, *A. cantonensis* lungworms have possibly spread across the southeastern United States. Our study highlights the importance of statewide or countrywide surveys to determine the full geographic distribution of *A. cantonensis* lungworms to inform strategies to mitigate the threat to both human and animal health.

Appendix Additional information for zoonotic rat lungworm *Angiostrongylus cantonensis* in black rats, Houston, Texas, 2024.
