# Supplementary material for: Zoonotic Rat Lungworm Angiostrongylus cantonensis in Black Rats, Houston, Texas, 2024
Source: Emerg Infect Dis. 2025 Sep;31(9):1857–60. doi: 10.3201/eid3109.251710 (PMC12407211; doi:10.3201/eid3109.251710)
Supplement: Appendix — Additional information for zoonotic rat lungworm Angiostrongylus cantonensis in black rats, Houston, Texas, 2024. [file 25-1710-Techapp-s1.pdf]

Article DOI: http://doi.org/10.3201/eid3109.251710

EID cannot ensure accessibility for supplementary materials supplied by authors. Readers who have difficulty accessing supplementary content should contact the authors for assistance.

Zoonotic Rat Lungworm *Angiostrongylus cantonensis* in Black Rats, Houston, Texas, 2024

Appendix

Methods

Between March and June 2024, 75 rodents were collected at a zoo in metropolitan Houston, Harris County, Texas (29.7158° N; 95.3903° W) either through poisoning or trapping. All rodents were identified as black rats based on morphological traits (e.g., relative tail length, size of eyes, shape of head). The specimens were stored frozen until processing via necropsy for evidence of *A. cantonensis.* Nematodes were manually recovered through dissection of the hearts and/or pulmonary vessels, counted and sexed by light microscopy, and stored in 70% ethanol. Lung tissues were then washed in water to allow the detection of intact or fragments of adult specimens, L1, or eggs in the resulting sediment. The right tibia of each rat was removed and measured as a proxy for age (*1*). Brain, heart, and lung tissues were fixed in 10% neutral buffered formalin.

Formalin-fixed tissues were processed and embedded in paraffin. Tissues were sectioned to 4 µm thickness and stained with hematoxylin and eosin.

Infected rats were identified from those collected in April−June 2024 (Appendix Figure). Representative male and female parasite specimens from each rat underwent DNA extraction, PCR, and sequencing. DNA was extracted from a 5–9 mm fragment of the anterior portion of the nematode using the DNeasy Blood and Tissue Kit for DNA Isolation (QIAGEN, https://www.qiagen.com/) according to manufacturer recommendations. PCR targeted a 200-bp segment of the cytochrome c oxidase subunit 1 gene (*cox1*). A 25 µL reaction containing 10µM of each of primer CO1ACF7 (5′-TGCCTGCTTTTGGGATTGTTAGAC-3′) and CO1ACR7 (5′-

TCACTCCCGTAGGAACCGCA-3′), 1X GoTaq Green Master Mix (Promega Corporation, https://www.promega.com), and 2.5 µL of DNA template was performed (*2*). Cycling parameters included: initial denaturation at 95°C for 2 min, then 40 cycles at 95°C for 30 s, 50°C for 30 s, 72°C for 90 s, and a final extension at 72°C for 5 min (*3*). Nuclease-free water served as a negative control, and DNA of *A. cantonensis* isolated from Georgia was used as a positive control. PCR products were purified using the EZNA Cycle Pure Kit (OMEGA Bio-Tek, https://www.omegabiotek.com) according to manufacturer instructions. Purified products were submitted to Eurofins Genomics for Sanger sequencing. Generated sequences were aligned and compared with sequences of *A. cantonensis* available in GenBank. Phylogenetic analysis was performed using MEGA X 10.2 (Figure 1) (*4*).

References

1. Rivory P, Bedoya-Pérez M, Ward MP, Šlapeta J. Older urban rats are infected with the zoonotic nematode *Angiostrongylus cantonensis.* Curr Res Parasitol Vector Borne Dis. 2024;5:100179. PubMed https://doi.org/10.1016/j.crpvbd.2024.100179

2. Qvarnstrom Y, Xayavong M, da Silva ACA, Park SY, Whelen AC, Calimlim PS, et al. Real-Time Polymerase Chain Reaction Detection of Angiostrongylus cantonensis DNA in Cerebrospinal Fluid from Patients with Eosinophilic Meningitis. Am J Trop Med Hyg. 2016;94:176–81. PubMed https://doi.org/10.4269/ajtmh.15-0146

3. Gottdenker NL, Nascimento Ramos RA, Hakimi H, McHale B, Rivera S, Miller BM, et al. *Angiostrongylus cantonensis* infection in brown rats (*Rattus norvegicus*), Atlanta, Georgia, USA, 2019–2022. Emerg Infect Dis. 2023;29:2167–70. PubMed https://doi.org/10.3201/eid2910.230706

4. Kumar S, Stecher G, Li M, Knyaz C, Tamura K. MEGA X: Molecular Evolutionary Genetics Analysis across Computing Platforms. Mol Biol Evol. 2018;35:1547–9. PubMed https://doi.org/10.1093/molbev/msy096

Appendix Table. Number of *Angiostrongylus cantonensis* intact specimens and fragment recovered from individual black rats (*Rattus rattus*) collected in Houston, Texas.

	Female			Male			Unknown Sex		
**Rat ID**	**Intact**	**Anterior fragment**	**Posterior fragment**	**Intact**	**Anterior fragment**	**Posterior fragment**	**Anterior fragment**	**Posterior fragment**	**Total**
**HRZ 006**	2	0	0	1	0	0	0	0	3
**HRZ 012**	0	2	0	0	1	0	0	0	3
**HRZ 020**	3	1	2	2	0	1	2	1	8
**HRZ 033**	0	2	1	3	0	1	2	1	7
**HRZ 037**	4	0	0	4	1	0	0	0	9
**HRZ 047**	44	0	4	33	0	1	0	0	82
**HRZ 055**	1	0	0	1	0	0	0	0	2
**HRZ 057**	28	0	1	18	0	0	0	0	47
**HRZ 062**	63	5	10	33	2	0	1	0	108
**HRZ 063**	36	2	0	24	0	0	0	0	62
**HRZ 067**	5	0	0	5	0	0	0	0	10
**HRZ 071**	26	0	0	12	0	0	0	0	38
**HRZ 072**	12	0	0	3	0	0	1	0	16
**HRZ 079**	1	0	0	1	0	0	0	0	2
**HRZ 080**	1	0	0	1	0	0	0	0	2

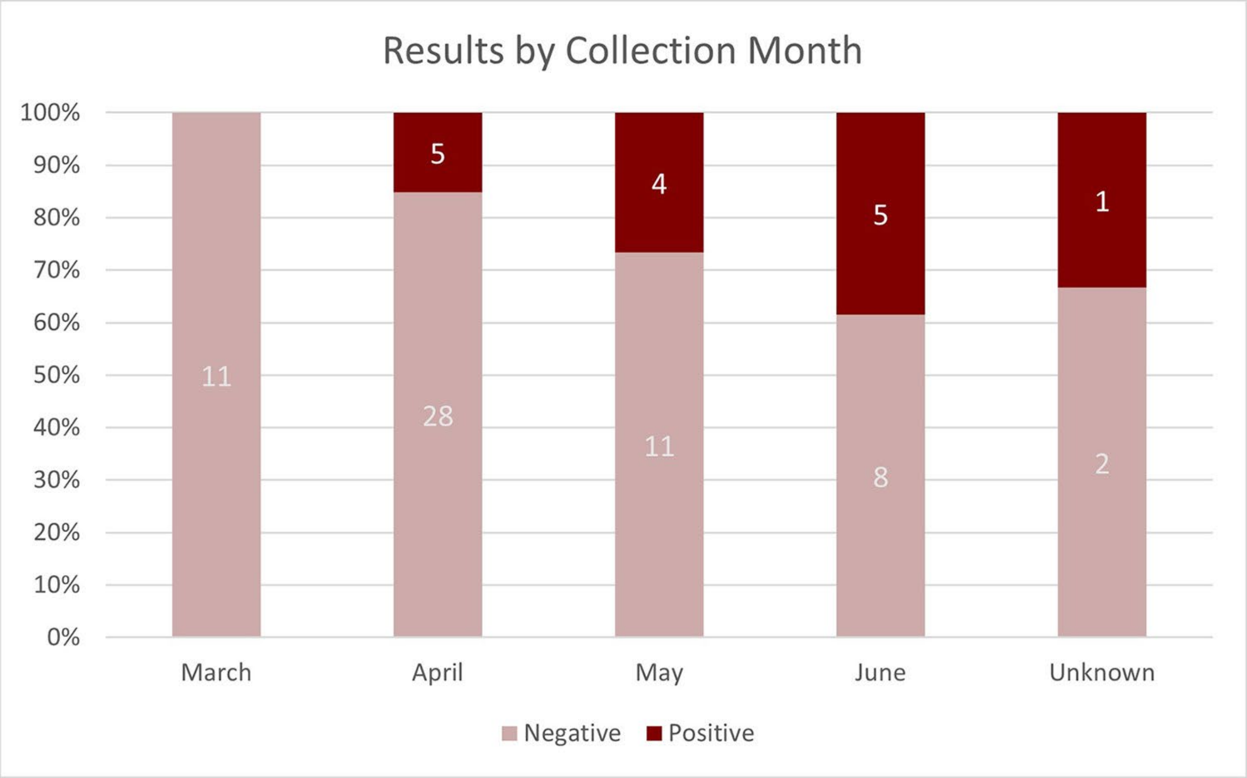

Appendix Figure. Number of black rats collected and confirmed to be infected with *Angiostrongylus cantonensis* by month of collection in a study of zoonotic rat lungworm in black rats, Houston, Texas, 2024.
